# Factors associated with older persons’ physical health in rural Uganda

**DOI:** 10.1371/journal.pone.0209262

**Published:** 2019-01-16

**Authors:** Fred Maniragaba, Abel Nzabona, John Bosco Asiimwe, Emmanuel Bizimungu, John Mushomi, James Ntozi, Betty Kwagala

**Affiliations:** 1 Department of Population Studies, Makerere University, Kampala, Uganda; 2 Department of Planning and Applied Statistics, Makerere University, Kampala, Uganda; 3 International Food Policy Research Institute, Kampala, Uganda; University of Malaya, MALAYSIA

## Abstract

**Introduction:**

The proportion of older persons in developing countries is increasing with no clear evidence of improvement in physical health. The aim of this paper was to examine the factors associated with older persons’ physical health in rural Uganda.

**Methods:**

This paper is based on a cross-sectional study of 912 older persons age 60 years and older across four major regions of Uganda. The study was conceptualized basing on World Health Organization quality of life BREF (WHOQOL-BREF). Analysis was done at three levels, that is, frequency distributions were generated to describe background characteristics of respondents and cross-tabulations were done to determine associations between dependent and each of the independent variables. Ordinal logistic regression was used to determine the predictors of physical health.

**Results:**

The likelihood of good physical health is high among older persons (Ops) who controlled their household assets (OR = 3.64; CI = 1.81–7.30) or the household assets controlled by their spouses (OR = 4.44; CI = 1.91–10.32) relative to those whose household assets were controlled by their children. There is high likelihood of good physical health among those who engage in physical activities (OR = 2.28; CI = 1.52–3.43) compared to those who do not.

**Conclusion:**

The findings have various policy implications, including creating an enabling environment and building capacities of older persons to remain in control of their household assets. Interventions focusing on deepening sensitization of older persons about importance of physical exercises could be a viable strategy for improving physical health of older persons.

## Introduction

Globally, human life expectancies have greatly improved with much progress happening in developing countries. It is projected that almost 80% of the world’s ageing population will be living in developing world [1]. The proportion of Africa’s older population is expected to increase from 5.1% in 2000 to 10.4% by 2050 [2, 3]. In sub-Saharan Africa, ageing population is taking place amidst challenges such as shortage of health infrastructure and services [4, 5]. This is worsened by the rise in non-communicable diseases such as cancer, cardiovascular diseases and several other later life health difficulties [6, 7]. Basing on the United Nations (UN) definition of older persons as those age 60 years and older [3], this study examines factors associated with physical health among older persons in rural Uganda.

Uganda’s population is largely characterized by young people. Nonetheless, the country’s older population is increasing steadily due to the high rate of survival and reduction in adult mortality [8, 9]. Estimated at 63 years, Uganda’s life expectancy has surpassed sub-Saharan Africa’s average of 60 years [9, 10]. The number of Uganda’s older persons age 60 years and above almost doubled between 1991 and 2002 (from 686,000 to 1.1 million persons)and increased to 1.6 million in 2014 [9]. Older persons constitute 4 percent of the total population [11]. The increase in ageing population is however against the backdrop of deterioration of social and family support systems that sustain the physical health and wellbeing of older persons [12]. For instance, rural-urban migration has drastically affected the care and support rendered to older persons [8]. Moreover, the existing social problems such as unemployment limit families’ capacities to support rural older persons’ healthcare [7, 8, 13].

Physical health dimension is one of the key indicators of people’s quality of life [14]. It includes activities of daily living, mobility status, fatigue; body pain, and sleep [15]. Poor physical health is the source of fear and discomfort among OPs thereby curtailing their productivity and reducing them to beggars and dependents [12]. Although research underscores the importance of physical health for OPs, this area remains understudied in Uganda [2, 11]. Existing literature about ageing population has mainly focused on loneliness [16], vulnerability of older adults [17], chronic poverty among elderly [18], nutritional status and functional ability of the elderly [19] and understanding the vulnerability of older adults [13]. Moreover, some of these studies used data covering a limited geographical scope and collected some years back. It is expected that the factors that influence physical health could have changed over time. Empirical evidence regarding factors influencing physical health among OPs is lacking. This research builds on earlier studies [13, 15, 17, 18] to bridge this knowledge gap. Using a recent empirical data from a wide geographical scope, we examine the factors influencing physical health of older persons in rural Uganda.

## Materials and methods

This paper utilized data from a cross sectional study on determinants of quality of life of 912 older men and women age 60 years and above in rural Uganda; representing a response rate (RR) of 95%. RR was calculated using the fourth formula from those recommended by the American association for Public Opinion Research [20]. The data were collected from February to March, 2017. Before data collection, enumerators with minimum qualification of a Bachelor’s degree were identified and recruited from respective study districts. These were trained on various aspects including research ethics, how to conduct interviews, and administering a questionnaire. Each interview took 35–45 minutes on average. During interviews, we made sure that we did not include persons who were sick. Multi-stage stratified cluster sampling approach was used. Uganda was stratified into four major regions namely; central, eastern, northern and western regions. Using simple random sampling, one district was selected from each of the four regions. In each district, three sub-counties were selected using simple random sampling. Four villages were selected using simple random sampling from each of the selected sub-counties, providing a total of 12 villages per district and hence 48 villages in the whole of Uganda. Using a community leader, a list of households with older persons was generated per village. Thereafter, ten households were randomly selected and older persons from 480 households were interviewed using an interviewer-administered questionnaire.

Physical health indicators were adapted from the World Health Organization Quality of Life (WHOQOL-BREF instrument). The WHOQOL-BREF is a World Health organization instrument that measures physical health, psychological health, social relationships, and environment [15, 21].It consists of 26 items, 24 of which are divided into four domains: physical health, mental health, social relationships, and environment. This study adapted with modifications the indicators of physical health dimension of quality of life. The items under this dimension include activities of daily living, mobility status, fatigue; body pain, and sleep [15]. The WHOQOL-BREF was preferred because it is flexible and is used on people from different cultures.

### Variables and measures

The dependent variable was physical health which was in form of an index. Independent variables were; age (60 years and older), sex (male or female), marital status (current marital status of respondent), region (region of residence), education level (education level acquired), religion (religion of respondent), type of house (whether house was permanent, semi-permanent or temporary), radio set ownership (whether respondent owned a radio or not), mobile phone ownership (whether respondent owned mobile phone or not), electricity (whether the respondent’s house had electricity or not), land ownership (whether responded owned land or not), control over household assets (who controlled household assets of the respondent), living arrangement (whether respondent lived alone or with others), financial support (whether respondent was financially supported or not), health care (who provided health care to respondent during sickness), distance to nearest health centre (distance of nearest health facility from respondent’s home in kilometers), HIV/AIDS sero-status (whether respondent was sero positive or sero negative), physical activity (whether respondent performed physical activities or not and fuel type (type of fuel used for cooking).

### Data analysis

Frequency distributions were computed to describe background characteristics of the respondents. Cross tabulations were done to determine the association between physical health and independent variables. A chi-square test statistic with a corresponding p-value was used to establish the significance of the association between the two variables. The level of statistical significance was fixed at 95% confidence (p = 0.05). Sampling weights were calculated to correct for imperfections resulting from selection of units with unequal probabilities.

We employed factor analysis (FA) to create an index for physical health using the 5 different indicators of; activities of daily living (whether respondent participated in activities of daily living or not), mobility status (whether respondent experienced mobility challenges or not), fatigue (whether respondent experienced fatigue or not); body pain (whether respondent experienced body pain or not), and sleep (whether respondent experienced any difficulties while sleeping or not). Factor loadings were rotated using quartimin oblique method for the loadings to reveal a clear pattern or the simple structure [22]. The selection of this rotation method was made based upon its popularity in use and availability in statistical software which seeks to minimize complexity only within the indicator variables [23] The technique was thus used to look for variables that factor well together but also with notable loading magnitude in absolute terms. High correlation among these indicators helped to produce a lower number of latent variables that fit common patterns in the data. Basing on the number of factors extracted, an index for the identified factors was calculated through linear combination between observed and factor loadings. Bartlett test of sphericity and the Kaiser-Meyer-Olkin (KMO) criterion [24] were performed to verify whether indicators in each category shared a common core. The Bartlett test was used to estimate the probability that the correlation matrix is zero, implying that all the variables are uncorrelated, while the KMO was used to indicate the extent to which variables had common feature to warrant factor analysis., KMO scores above 0.5 (threshold scores) were acceptable; scores above 0.9 were exceptional [25]. In this study, the analysis yielded a KMO value of 0.814, while the Bartlett test yielded $\chi_{\text{253}}^{2}$ = 7057.335 (*p = 0*.*000*), signifying the data’s adequacy for factor analysis.

Since the outcome variable formed through FA was in form of index, the study objective was achieved by use of the ordered logistic regression model. The dependent variable (Physical health) was grouped into three categories. That is, good, fair and poor basing on the range with which a respondent’s score laid. In this dimension of quality of life used in this study, individuals are expected to have varying levels of physical health with some better off or worse off than others. The strengths of the relationships were reported as odds ratios. The p-value was used to determine whether the relationship was significant or not depending on the level of significance which was fixed at 0.05.

### Ethical considerations

Before data collection, ethical clearance was granted by National HIV/AIDS research committee (NARC, Ref: ARC 190), Uganda National Council for Science and Technology (UNCST, Ref: SS 4167) and Office of the President of the Republic of Uganda (Ref: ADM 194/212/01). While collecting data, voluntary written informed consent was obtained from all respondents prior to the interviews and participants were assured of confidentiality. All information was provided in the relevant local languages (Rukiga, Langi, Luganda and Lusoga).

## Results and discussion

### Descriptive characteristics of the older persons

Table 1 shows that the proportion of older persons reduced with advancement in age. The majority of the respondents (51%) were age 60–69 years. More than half of the respondents (57%) were females. Although five in every ten respondents (51%) were currently married, the level of widowhood was also noticeably high (40%). Regions were proportionately represented with central having the highest proportion (27%). More than a half (54%) had no formal education and 40% had primary education. The largest proportion (38%) of the respondents were affiliated to the Anglican faith, followed by Catholic (37%) and Muslims (13%); 69% were subsistence farmers by occupation. Over two-fifths (41%) were staying in permanent houses; 52% possessed radios; nearly three-quarters (73%) did not have mobile phones; 91% did not have electricity in for lighting in their houses; and 91% owned land for both agriculture and settlement. Majority of the respondents (76%) controlled their household resources and 74% lived with their children and grandchildren. Over half (56%) were not receiving financial support; 41% were getting healthcare support from their children; 48% travelled for 1–2 kilometers to reach the nearest health center; 97% were HIV sero negative; nearly three-quarters (74%) were engaging in physical activity; and 96% were using firewood as the main form of fuel for cooking. Respondents in the categories of physical health namely; good, fair and poor were equally represented (33%) as seen in Table 1.

**Table 1:** Percent distribution of respondents by selected socio-demographic characteristics.

Variable	Number	Percent (%)
**Age**		
60–69	462	50.7
70–79	290	31.8
80+	160	17.5
**Sex**		
Male	395	43.3
Female	517	56.7
**Marital status**		
Married	461	50.6
Widowed	361	39.5
Divorced/Separated	90	9.9
**Region**		
Central	248	27.2
East	237	25.9
North	190	20.8
West	237	25.9
**Education level**		
No education	495	54.3
Primary	363	39.8
Secondary	31	3.4
Tertiary	23	2.5
**Religion**		
Catholic	342	37.5
Anglican	350	38.4
Muslim	118	12.9
Pentecostal	71	7.8
SDA	20	2.2
Other	11	1.2
**Occupation**		
Farming	627	68.8
Others	23	2.5
None	262	28.7
**Type of house**		
permanent	369	40.5
Semi-permanent	320	35.1
Temporary	223	24.4
**Radio set ownership**		
Yes	479	52.5
No	433	47.5
**Mobile phone ownership**		
Yes	251	27.5
No	661	72.5
**Has electricity**		
Yes	90	9.9
No	822	90.1
**Land ownership**		
Yes	825	90.5
No	87	9.5
**Control Over household assets**		
Self	693	76.0
Spouse	132	14.5
children	87	9.5
**Living arrangement**		
Alone	80	8.8
Spouse only	22	2.4
Spouse & Children	133	14.6
Children & Grandchildren	677	74.2
**Financial support**		
Yes	404	44.3
No	508	55.7
**Healthcare in sickness**		
Spouse	308	33.8
Children	377	41.3
Grandchildren	91	10.0
Others	136	14.9
**Distance to the nearest health center**		
0–0.5 Km	170	18.6
1–2 Km	433	47.5
> 2 Km	309	33.9
**Has HIV/AIDS**		
Yes	28	3.2
No	854	96.8
**Do physical activities**		
Yes	678	74.4
No	233	25.6
**Fuel type**		
Fire wood	871	95.5
Charcoal	41	4.5
**Physical health**		
Poor	304	33.3
Fair	304	33.3
Good	304	33.3

### Association between physical health and demographic and socio-economic factors

Table 2 presents chi-square test results for the association between physical health and selected demographic, socio-economic and social support factors. Religion, type of residential house, financial support, distance to the nearest health facility, HIV sero status and type of fuel for cooking were not significantly associated with physical health.

**Table 2:** Percentages of older persons by selected factors affecting their physical health.

Variable	Physical health			Number (n = 912)	*χ*^2^	*p-value*
	Poor (%)	Fair (%)	Good (%)			
**Age**						
60–69	22.3	36.4	41.3	462		
70–79	35.9	31.0	33.1	290	85.2	**<0.001**
80+	58.8	30.6	10.6	160		
**Sex**						
Male	28.1	32.4	39.5	395		
Female	37.3	34.0	28.6	517	13.8	**0.001**
**Region**						
Central	35.5	45.2	19.3	248		
East	33.3	26.2	40.5	237	51.1	**<0.001**
North	29.5	40.0	30.5	190		
West	34.2	22.8	43.0	237		
**Education level**						
No education	37.9	35.9	26.1	495		
Primary	29.5	30.9	39.6	363	33.9	**<0.001**
Secondary	16.1	29.3	54.9	31		
Tertiary	17.4	21.7	60.9	23		
**Marital status**						
Married	28.2	30.8	41.0	461	28.0	**<0.001**
Widowed	40.2	35.7	24.1	361		
Divorced	32.2	36.7	31.1	90		
**Religion**						
Catholic	34.8	32.5	32.7	342		
Anglican	30.9	34.6	34.5	350		
Muslim	28.0	41.5	30.5	118		
Pentecostal	35.2	26.8	38.0	71	18.2	0.051
SDA	60.0	15.0	25.0	20		
Others	63.6	9.1	27.3	11		
**Occupation**						
Farming	24.4	36.8	38.8	627		
Other	17.4	34.8	47.8	23	88.5	<**0.001**
None	56.1	24.8	19.1	262		
**Type of house**						
Permanent	36.8	31.8	31.4	223		
Semi-permanent	32.2	30.0	37.8	320	6.8	0.144
Temporary	32.3	37.1	30.6	369		
**Radio set ownership**						
Yes	25.7	32.6	41.8	479		
No	41.8	34.2	24.0	433	39.4	**<0.001**
**Mobile phone ownership**						
Yes	25.1	28.7	46.2	251		
No	36.4	35.1	28.4	661	26.5	**<0.001**
**Has electricity**						
Yes	32.2	17.8	50.0	90		
No	33.5	35.0	31.5	822	15.6	**<0.001**
**Land ownership**						
Yes	31.9	33.3	34.8	825		
No	47.1	33.3	19.5	87	11.0	**0.004**
**Control over household assets**						
Self	28.3	35.8	35.1	693		
Spouse	32.6	28.8	38.6	132		
Children	74.7	19.5	5.8	87	79.4	**<0.001**
**Living arrangement**						
Alone	36.3	37.5	26.2	80		
Spouse only	31.8	27.3	40.9	22		
Spouse & children	25.6	27.8	46.6	133	14.4	**0.026**
Children & grandchildren	34.6	34.1	31.3	677		
**Financial support**						
Yes	35.6	32.4	31.9	404		
No	31.5	34.1	33.4	508	1.8	0.413
**Healthcare in sickness**						
Spouse	18.8	33.1	48.1	308		
Children	43.5	31.3	25.2	377	64.5	**<0.001**
Grandchildren	32.9	40.7	26.4	91		
Others	38.2	34.6	27.2	136		
**Distance to the nearest health center**						
0–0.5 Km	33.5	31.8	34.7	170		
1–2 Km	33.9	31.9	34.2	426	1.9	0.760
> 2 Km	32.4	36.3	31.4	309		
**Has HIV/AIDS**						
Yes	32.1	28.6	39.3	28		
No	33.9	33.4	32.7	854	0.6	0.750
**Do physical activities**						
Yes	25.2	36.4	38.4	678		
No	56.6	24.5	18.9	233	78.6	**<0.001**
**Fuel type**						
Fire wood	33.3	33.2	33.5	871		
Charcoal	34.1	36.6	29.3	41	0.4	0.836

p = Pearson χ^2^ test

The results show that the prevalence of self-reported poor physical health among older persons was highest among the oldest old age 80+ (59%; p < 0.001), women (37%; p<0.001), those living in central region (36%; p<0.001), those with no formal education (40%; p<0.001), widowed (40%; p<0.001), no occupation (56%; p<0.001), those with no radio set ownership (42%; p<0.001), no mobile phone (36%; p<0.001), no electricity for lighting in their houses (34%; p<0.001), no land ownership (47%; p = 0.004). Furthermore, self-reported poor physical health was highest among older persons whose children controlled their household assets (75%; p<0.001), those who were living alone (36%; p = 0.026), those whose healthcare was derived from their children (44%; p<0.001) and not doing physical activity (57%; p<0.001).

### Predictors of physical health

Results of the ordered logistic regression model of factors influencing physical health of older persons are presented in Table 3. The results show that adjusting for other variables, the odds of good versus fair and poor physical health reduced with advancement in age. Compared to ages 60–69, the likelihood of having poor physical health was higher among older persons age 70–79 years (OR = 0.688; p = 0.026) and those age 80 years and older (OR = 0.349; p**<**0.001**)**. The findings also show that male older persons had increased odds (OR = 11.549; p = 0.022) of having good physical health compared to their female counterparts. Living in eastern region was associated with good physical health (OR = 1.900; p = 0.001) compared to living in the central region. The Results further indicate that older persons who possessed a radio set were nearly two times more likely to have good physical health (OR = 1.928; p<0.001) compared to those who did not. Similarly, compared to the older persons whose household assets were controlled by their children, those who controlled their household assets were more likely to have good physical health (OR = 2.292; p = 0.008). Table 3 also shows that older persons whose healthcare support was derived from their spouses were more likely to have poor physical health (0.468; p = 0.001) compared to those who obtained healthcare support from their children. Older persons that engaged in physical activity were two times (OR = 2.375; p = <0.001) more likely to have good physical health than those who were not.

**Table 3:** Results of ordered logistic regression of the predictors of physical health of older persons.

Variable	Odds ratio	95% CI	*p-value*
**Age**			
60–69*	1.000		
70–79	0.688	[0.494–0.957]	**0.026**
80+	0.349	[0.226–0.541]	**<0.001**
**Sex**			
Male	1.549	[1.064–2.258]	**0.022**
Female*	1.000		
**Region**			
Central*	1.000		
East	1.900	[1.281–2.821]	**0.001**
North	1.074	[0.645–1.789]	0.782
West	1.089	[0.669–1.773]	0.731
**Education level**			
No education*	1.000		
Primary	0.868	[0.626–1.203]	0.394
Secondary	1.541	[0.603–3.937]	0.366
Tertiary	1.587	[0.516–4.886]	0.420
**Marital status**			
Married*	1.000		
Widowed	1.479	[0.860–2.545]	0.157
Divorced	1.711	[0.899–2.254]	0.101
**Type of house**			
Temporary*	1.000		
Semi-permanent	1.124	[0.759–1.663]	0.560
Permanent	0.674	[0.432–1.052]	0.082
**Radio set ownership**			
Yes	1.928	[1.400–2.656]	**<0.001**
No*	1.000		
**Has electricity in the house**			
Yes	1.288	[0.651–2.546]	0.467
No*	1.000		
**Land ownership**			
Yes	1.308	[0.792–2.158]	0.294
No*	1.000		
**Control over household assets**			
Self	2.292	[1.239–4.239]	**0.008**
Spouse	2.043	[0.936–4.461]	**0.073**
Children*	1.000		
**Living arrangement**			
Alone*	1.000		
Spouse only	1.228	[0.353–4.271]	0.747
Spouse & Children	0.862	[0.391–1.899]	0.712
Children & Grandchildren	0.686	[0.479–1.512]	0.583
**Material support**			
Yes	0.790	[0.585–1.067]	0.124
No*	1.000		
**Healthcare in sickness**			
Spouse*	1.000		
Children	0. 468	[0.293–0.745]	**0.001**
Grandchildren	0. 702	[0.382–1.291]	0.255
Others	0. 686	[0.347–1.358]	0.279
**Has HIV/AIDS**			
Yes	0.857	[0.332–2.209]	0.749
No*	1.000		
**Do physical activity**			
Yes	2.375	[1.583–3.562]	**<0.001**
No*	1.000		
**Fuel type**			
Firewood*	1.000		
Charcoal	0.819	[0.403–1.669]	0.584

*Reference category

## Discussion

Our findings show that poor physical health was associated with advanced age especially among older persons age 80 years and older. This finding aligns with findings of studies done in Tanzania [26], Brazil [27], England [28], South Africa [29], Sweden [30] and other countries [31–35] which reveal that as people advance in age, they become frail and begin to experience frequent falls, functional complications and lead a dependency life in activities of daily living such as bathing, toileting, eating and walking.

This study found that male older persons were more likely to have good physical health compared to their female counterparts. Whereas our finding indicates that older male persons are more likely to be physically healthy than women, literature concerning mortality rates of older persons in Uganda is generally unavailable. However, available literature from Uganda National Population and Housing Census of 2014 indicates that the life expectancy for women in Uganda is higher (64.2 years) compared to 62.2 for men [9]. This contradicts our finding which seems to suggest that physical health may not be correlated with life expectancy. However, in conformity with this finding, previous research has shown that older women experience functional disabilities than men [27, 29]. This finding could also be attributed to gender imbalances in access to and control over socio-economic and political resources where men are more advantaged than women [31, 36, 37].

Regarding region, our findings indicate that older persons living in eastern region were more likely to have good physical health compared to those in central region. Regional disparities existing in OPs’ physical health could be resulting from cultural differences and life styles especially in diet and work status as well as disparities in the availability and accessibility to health services and healthcare support. This finding is in consonance with other studies [38–41] which show that the environment or context within which OPs live affects their health.

The odds of good physical health increased among older persons who owned a radio set. It could be argued that radios perhaps facilitate in dissemination of health information to the older persons since some of the programmes are educative in health matters. In addition, they address loneliness through entertainment. This finding aligns with previous studies which found that radio was one of the reliable sources of information in health related issues of older persons [42].

OPs whose assets were controlled by themselves or their spouses were more likely to have good physical health compared to their counterparts whose assets were controlled by their children. This could partly be because having control over assets leads to quicker decision making on matters relating to better health for the older person. This result is in agreement with previous studies [31, 43–46] which show that older persons who possess assets are more likely to generate incomes that facilitate access to healthcare and other social services that enhance their physical health [31, 43–46]. Similarly, older persons whose health care was supported by their children had poor physical health. Co-residence is currently rare making it difficult for children to provide quality care to their parents [47, 48]. Previous studies [8, 13, 18, 48–50] show that low healthcare support to the older persons by children results from family disruptions such as increasing rate of nuclear families education and rural-urban migration where children choose to live far away from their ageing parents with limited support.

The findings indicate that older persons who engaged in physical activity were more likely to have good physical health. In agreement with the previous studies [51–55], physical activity strengthens health status of older persons through decreased falls and morbidity. The major limitation of this study is that analysis relied on self-reported responses and not from anthropometric measures. Inadequate funding could not enable us to do objective analysis. Thus, the responses could have been under reported, over reported or misreported. Another limitation is that this study did not use complex survey analysis that would adjust for cluster sampling.

## Conclusions

The odds of poor physical health were higher among; OPs of advanced age, females, those who lived in central region of the country, those who did not own a radio, those whose children controlled household assets and supported healthcare. Similarly, the OPs who did not engage in physical activity were more likely to have poor physical health. These findings have various policy implications, including creating an enabling environment and building capacities of older persons to remain in control of their household assets. Interventions focusing on deepening sensitization of older persons about importance of physical exercises could be a viable strategy for improving physical health of older persons.

## Supporting information

S1 Table Summary of rotated factor loadings against indicators (n = 912). (DOCX)

S1 File Questionnaire. (DOCX)

S2 File Physical health dataset. (DTA)
