# Supplementary material for: Factors associated with older persons’ physical health in rural Uganda
Source: PLoS One. 2019 Jan 16;14(1):e0209262. doi: 10.1371/journal.pone.0209262 (PMC6334921; doi:10.1371/journal.pone.0209262)
Supplement: S1 Table — (DOCX) [file pone.0209262.s001.docx]

**Appendix I: Summary of rotated factor loadings against indicators (n = 912)**

|  | Factor 1  ‘Social participation’ | Factor 2  ‘Physical health’ | Factor 3 ‘  Intimacy’ |
| --- | --- | --- | --- |
| Participates in religious functions | **0.8564** | 0.0133 | -0.0311 |
| Participates in elderly clubs | 0.2961 | 0.1555 | -0.0276 |
| Participates in local ceremonies | **0.6398** | -0.0891 | 0.0591 |
| Participates in visiting friends | **0.7773** | 0.0473 | 0.0368 |
| Participates in charity walks | 0.2099 | 0.2214 | 0.0472 |
| Participates in social gatherings | 0.4601 | -0.0571 | 0.1289 |
| Participates in community meetings | **0.7318** | 0.0402 | 0.0280 |
| Participates in burial/funeral | **0.8561** | -0.0253 | -0.0329 |
| I always have difficulties in hearing | 0.1013 | 0.3696 | -0.0645 |
| I always have difficulties in seeing | 0.0848 | 0.4653 | 0.0672 |
| I always have difficulties in moving on my own | - -0.0077 | **0.6030** | - -0.0549 |
| I always have difficulties with feeding myself | 0.0330 | 0.3680 | -0.4193 |
| I always have difficulties with taking a bath or shower | 0.1223 | 0.4249 | -0.2752 |
| I always have difficulties while dressing | -0.0025 | 0.4288 | -0.4023 |
| I have been with body pain in the past one month | -0.0276 | 0.4417 | 0.1816 |
| I have experienced pain around the joints in past 3 months | -0.0899 | **0.5809** | 0.1218 |
| I easily get tired even when I have not done much work | -0.1049 | **0.5031** | 0.1418 |
| I have enough energy for everyday life | 0.1473 | -0.4193 | -0.0977 |
| I always have difficulties in sleeping | 0.0081 | **0.5644** | 0.0065 |
| I have opportunities to love other people | 0.0885 | 0.0618 | **0.6107** |
| I am loved by other people around me | 0.0635 | 0.0811 | **0.7105** |
| I live happily with my family members | -0.0593 | 0.0057 | **0.6169** |
| I no longer engage in sexual relations | -0.1988 | 0.1723 | 0.1497 |
