# Supplementary material for: Factors associated with older persons’ physical health in rural Uganda
Source: PLoS One. 2019 Jan 16;14(1):e0209262. doi: 10.1371/journal.pone.0209262 (PMC6334921; doi:10.1371/journal.pone.0209262)
Supplement: S1 File — (DOCX) [file pone.0209262.s002.docx]

# Appendix 11: Questionnaire

**SECTION A: IDENTIFICATION AND BASIC INFORMATION**

| **SURVEY INFORMATION:**   \| Survey staff \| Name \| Staff ID \| Date completed \| \| \| \| --- \| --- \| --- \| --- \| --- \| --- \| \| Day \| Month \| Year \| \| 1. Interviewer \|  \|  \|  \|  \| 2016 \| \| 1. Supervisor \|  \|  \|  \|  \| 2016 \| \| 1. Principal Researcher \|  \|  \|  \|  \| 2016 \| \| 1. Data Entrant \|  \|  \|  \|  \| 2016 \| | **LOCATION IDENTIFIERS:**   \| LOCATION \| . Name \| *LOCATION-Code* \| \| --- \| --- \| --- \| \| 1. Region \|  \|  \| \| 1. District \|  \|  \| \| 1. Sub county \|  \|  \| \| 1. Parish \|  \|  \| \| 1. Village \|  \|  \| |
| --- | --- | --- | --- | --- | --- | --- | --- | --- | --- | --- | --- | --- | --- | --- | --- | --- | --- | --- | --- | --- | --- | --- | --- | --- | --- | --- | --- | --- | --- | --- | --- | --- | --- | --- | --- | --- | --- | --- | --- | --- | --- | --- | --- | --- | --- | --- | --- | --- | --- | --- | --- | --- |

**SECTION 100: INDIVIDUAL CHARACTERISTICS OF THE RESPONDENT**

**Now, I would like to ask you some questions concerning yourself in general.**

| **Question** | **Question** | **Questions** |
| --- | --- | --- |
| **101**. Sex (***observe and record***)   \| 1. Male \|  \| \| --- \| --- \| \| 2. Female \|  \| | **102**. When were you born?   \| Year \|  \| \| --- \| --- \| \| Don’t know *(****Enter 99***) \|  \| | \| **103**. What is your age (***in complete years***) \|  \| \| --- \| --- \|   (check for consistency between 102 and 103)  **If less than 60, do not proceed with the interview** |
| **104**. Have you ever attended school?   \| 1. Yes \|  \|  \| \| --- \| --- \| --- \| \| 2. No \|  \| (***skip to Q106***) \| | **105**. What is the highest level of formal education you completed?   \| 1. Primary \|  \| 3. Tertiary \|  \| \| --- \| --- \| --- \| --- \| \| 2. Secondary \|  \| 4. University \|  \| | **106**. What is your religion?   \| 1. Catholic \|  \| 4. Pentecostal \|  \| \| --- \| --- \| --- \| --- \| \| 2. Anglican \|  \| 5. SDA \|  \| \| 3. Muslim \|  \| 88. Others(Specify) \|  \| \|  \|  \|  \|  \| |
| **107**. What is your current marital status?   \| 1. Never Married \|  \| 4. Widowed \|  \| \| --- \| --- \| --- \| --- \| \| 2. Married \|  \| 5. Divorced \|  \| \| 3. Cohabiting \|  \| 6. Separated \|  \| | **108**. What is your ***primary*** source of money?   \| 1. Savings \|  \| 5. Family \|  \| \| --- \| --- \| --- \| --- \| \| 2. Farming \|  \| 6. Friends \|  \| \| 3. Wage labour \|  \| 88. Others (specify) \|  \| \| 4. Social security \|  \|  \|  \| |  |

**SECTION 200: HOUSEHOLD CHARACTERISTICS**

*Now, I want us to discuss conditions in this household:*

| **Question** | **Question** | **Question** |
| --- | --- | --- |
| 201. What type of fuel does your household use mainly for cooking?   \| 1. Firewood \|  \| \| --- \| --- \| \| 2. Charcoal \|  \| \| 3. Paraffin \|  \| \| 4. Electricity \|  \| \| 5. Biogas \|  \| \| 88. Others (specify) \|  \| | 202. If firewood, who fetches it for you?   \| 1. Self \|  \| \| --- \| --- \| \| 2. Spouse \|  \| \| 3.Children \|  \| \| 4.Grandchildren \|  \| \| 5. Relatives \|  \| \| 6. House workers \|  \| \| 88. Others (specify) \|  \| | 203. What source of energy does this household mainly use for lighting?   \| 1. Electricity (UMEME) \|  \| \| --- \| --- \| \| 2. Solar electricity \|  \| \| 3. Generator \|  \| \| 3. Biogas \|  \| \| 4. Tadooba \|  \| \| 5. Firewood \|  \| \| 6. Candles \|  \| \| 88. Others(specify) \|  \| |
| **204**. What is the household’s main source of water?   \| 1. Piped water \|  \| \| --- \| --- \| \| 2. Protected well/spring \|  \| \| 3. Unprotected well/spring \|  \| \| 3. Borehole \|  \| \| 4. Rain water \|  \| \| 5. River/lake/stream \|  \| \| 88. Others(specify) \|  \| \|  \|  \| | **205**. What is the distance to this source of water from home?   \| 1. < 0.5 km \|  \| \| --- \| --- \| \| - 1. 0.5 km \|  \| \| 2. 1 Km \|  \| \| 3. 2 Km \|  \| \| 4. >2 Km \|  \| | **206**. Who fetches water from the source for you?   \| 1. Self \|  \| \| --- \| --- \| \| 2. Spouse \|  \| \| 3.Children \|  \| \| 4.Grandchildren \|  \| \| 5. Relatives \|  \| \| 6. House workers \|  \| \| 88. Others (specify) \|  \| |
| 207. What type of a toilet facility does your household mainly use?   \| 1. Flush toilet \|  \| \| --- \| --- \| \| 2. Covered pit latrine \|  \| \| 3. Uncovered pit latrine \|  \| \| 4. Bush \|  \| \| 88. Others (specify) \|  \| | **208**. What is your household’s main source of information   \| 1. Radio \|  \| \| --- \| --- \| \| 2. Word of mouth \|  \| \| 3. Telephone \|  \| \| 4. Print media \|  \| \| 5. Television \|  \| \| 6. Community meetings \|  \| \| 88. Others(specify) \|  \| \|  \|  \| | \|  \|  \|  \| \| --- \| --- \| --- \| \| **209**. Generally, how many people live in your household \| \| \| \|  \| |
| **2010**. Of these, how many are:   \| - 1. Spouses \|  \| 4. Other relatives \|  \| \| --- \| --- \| --- \| --- \| \| 2. Own children \|  \| 6. House workers \|  \| \| 3. Grandchildren \|  \| 88. Others(specify) \|  \| | **211**. Who controls money in this household   \| 1. Self \|  \| \| --- \| --- \| \| 2. Spouse \|  \| \| 3. Child(ren) \|  \| \| 4. Another family member \|  \| \| 5.Non family member \|  \|   (***Only one response expected***) | **212**. Do you have these items in your household?   \| 1. Electricity \|  \| \| --- \| --- \| \| 2. Radio \|  \| \| 3. Television (TV) \|  \| \| 4. Mobile phone \|  \| \| 5. Car \|  \| \| 6.Motocycle \|  \| \| 6.Bicycle \|  \| \| 88.Others, (specify) \|  \| |
| 213. What type of a house do you occupy   \| 1. Detached house \|  \| \| --- \| --- \| \| 2. Apartment \|  \| \| 3. Tenement \|  \| \| 88.Others (specify) \|  \| | 214. What is the quality of your house in terms of durability?   \| 1. Permanent \|  \| \| --- \| --- \| \| 2. Semi-permanent \|  \| \| 3. Temporary \|  \| \| 4.Delapidated \|  \| \| 5. Make shift \|  \| \| 88. Others (specify) \|  \| | **215**. Do you owns the house you currently occupy?   \| 1. Yes \|  \| (***skip to Q216***) \| \| --- \| --- \| --- \| \| 2. No \|  \|  \| |
| **216**. Who bought/constructed the house you currently occupy?   \| 1. Spouse \|  \| \| --- \| --- \| \| 2. Child \|  \| \| 2. Relatives \|  \| \| 88. Others (specify) \|  \| | **217**. Does this household own any animals or birds?   \| 1. Yes \|  \|  \| \| --- \| --- \| --- \| \| 2. No \|  \| (***skip to Q221)*** \| | **218**. Of the animals and birds owned, how many are:   \| 1. Cows \|  \| \| --- \| --- \| \| 2. Goats \|  \| \| 3. Sheep \|  \| \| 4. Pigs \|  \| \| 5. Chicken \|  \| \| 6. Donkeys \|  \| \| 88. Others (specify) \|  \| |
| **219**. Do you own land for either agriculture or settlement?   \| 1. Yes \|  \|  \| \| --- \| --- \| --- \| \| 2. No \|  \| (***skip to Q220***) \| | **220**. Who mainly tills land where you do farming?   \| 1. Self \|  \| \| --- \| --- \| \| 2.Spouse \|  \| \| 3. Children/Grandchildren \|  \| \| 4. Relatives \|  \| \| 5. Hired laborers \|  \| \| 6. Neighbors \|  \| \| 88. Others(specify) \|  \| \|  \|  \| | **221**. Overall, who controls assets in this household?   \| 1. Self \|  \| \| --- \| --- \| \| 2. Spouse \|  \| \| 3. Children \|  \| \| 88. Others(specify) \|  \| \|  \|  \| |

**SECTION 300: NETWORKS AND SOCIAL SUPPORT**

Now, I want us to discuss you networks, relatives’ social relationships

| **Question** | **Question** | **Question** |
| --- | --- | --- |
| 301. Do you live with some of your children?   \| 1. Yes \|  \|  \| \| --- \| --- \| --- \| \| 2. No \|  \| ***Skip to Q303*** \| | 302. How do you keep in touch with the children you stay with?   \| 1. Phone \|  \| \| --- \| --- \| \| 2. Letter \|  \| \| 3. They visit me \|  \| \| 4. I visit them \|  \| \| 88. Others (specify) \|  \| | 303. Do some of your children live elsewhere in Uganda or in other countries?   \| 1. Yes \|  \|  \| \| --- \| --- \| --- \| \| 2. No \|  \| ***Skip to Q306*** \| |
| 304. Where do your children living elsewhere live?   \| 1. Rural Uganda \|  \| \| --- \| --- \| \| 2. Urban Uganda \|  \| \| 3. Outside Uganda \|  \| | 305. How do you keep in touch with your children who live elsewhere in Uganda or in other countries?   \| 1. Phone \|  \| \| --- \| --- \| \| 2. Letter \|  \| \| 3. They visit me \|  \| \| 4. I visit them \|  \| \| 88. Others (specify) \|  \| | 306. Regardless where they live, do your children support you financially?   \| 1. Yes \|  \|  \| \| --- \| --- \| --- \| \| 2. No \|  \| ***Skip to Q309*** \| |
| 307. How often do your children give you financial support?   \| 1. Daily \|  \| 4. Quarterly \|  \| \| --- \| --- \| --- \| --- \| \| - 1. Weekly \|  \| 5. Annually \|  \| \| 3. Monthly \|  \| 88. Other (specify) \|  \| \|  \|  \|  \|  \| | 308. How do you rate the financial support you receive from your children   \| 1. Adequate \|  \| \| --- \| --- \| \| 2. Inadequate \|  \| | 309. Other than your children, do you have other relatives and friends who live in other parts of the country or outside the country?   \| 1. Yes \|  \|  \| \| --- \| --- \| --- \| \| 2. No \|  \| ***Skip to Q312*** \| |
| 310. What is the method of keeping in touch with them?   \| 1. Letter \|  \| \| --- \| --- \| \| 2. Phone \|  \| \| 3. They visit me \|  \| \| 4. I visit them \|  \| \| 88. Others (specify) \|  \| | 311. Do you get material support from them?   \| 1. Yes \|  \| \| --- \| --- \| \| 2. No \|  \| | 312. Who supports you materially?   \| 1. Spouse \|  \| \| --- \| --- \| \| 2. Children \|  \| \| 3. Relatives \|  \| \| 4. Neigbours \|  \| \| 5. Friends \|  \| \| 6.None \|  \| \| 88. Others (specify) \|  \| |
| 313. Who of these people do you always share with your personal matters or any other issue?   \| 1. Spouse \|  \| \| --- \| --- \| \| 2. Children \|  \| \| 3. Relatives \|  \| \| 4. Neigbours \|  \| \| 5. Friends \|  \| \| 6.None \|  \| \| 88. Others (specify) \|  \| | 314. Who looks after you at home when you are sick?   \| 1. Spouse \|  \| \| --- \| --- \| \| 2. Children \|  \| \| 3. Relatives \|  \| \| 4. Neigbours \|  \| \| 5. Friends \|  \| \| 6.None \|  \| \| 88. Others (specify) \|  \| | 315. Who accompanies you to the hospital or health centre when you are sick?   \| 1. Spouse \|  \| \| --- \| --- \| \| 2. Children \|  \| \| 3. Relative \|  \| \| 4. Friend \|  \| \| 5. Neigbour \|  \| \| 88. Others (specify) \|  \| |
| 316. What is the distance to the nearest health centre from home?   \| 1. < 0.5 km \|  \| \| --- \| --- \| \| - 1. 0.5 km \|  \| \| 2. 1 Km \|  \| \| 3. 2 Km \|  \| \| 4. >2 Km \|  \| | 317. Who travels to the market or shop to buy items you need?   \| 1. Self \|  \| \| --- \| --- \| \| 2. Spouse \|  \| \| 3. Children \|  \| \| 4. Relative \|  \| \| 5. Friend \|  \| \| 6. Neigbour \|  \| \| 7. House worker \|  \| \| 88. Others (specify) \|  \| | 318. Who assists you in household chores like cooking, washing, sweeping etc.?   \| 1. Self \|  \| \| --- \| --- \| \| 2. Spouse \|  \| \| 3. Children \|  \| \| 4. Relatives \|  \| \| 5. Friends \|  \| \| 6. Neigbours \|  \| \| 7. House worker \|  \| \| 88. Others (specify) \|  \| |
| 319. Do you have friends   \| 1. Yes \|  \|  \| \| --- \| --- \| --- \| \| 2. No \|  \| ***Skip to Q324*** \| | 320. How many friends do you have?   \|  \| \| --- \| | 321. Are you satisfied with the number of friends you have?   \| 1. Yes \|  \| \| --- \| --- \| \| 2. No \|  \| |
| 322. How do you get along with your friends? | 323. How important is it to have many friends? | 324. Are you satisfied with your relationship with your family?   \| 1. Yes \|  \| \| --- \| --- \| \| 2. No \|  \| |
| 325. How important are family relationships to you? | 326. Do you have neighbours?   \| 1. Yes \|  \|  \| \| --- \| --- \| --- \| \| 2. No \|  \| **Skip to Q328** \| | 327. How many neighbours do you have |
| 328 How important is your neighborhood as a place for your family to live in? | 329. Are you a member of any organization/SACCO?   \| 1. Yes \|  \|  \| \| --- \| --- \| --- \| \| 2. No \|  \| **Skip to Q 401** \| | 330. In which organization/SACCO do you hold membership? |
| 331. What is your position in that organization/SACCO? | 332. What activities do these organization/SACCOs do? | 333. How do you benefit from these organizations/SACCOs? |

**SECTION 400: SOCIAL ACTIVITY**

**Now I want to ask you about social activity you engage in**

| **Question** | **Question** | **Question** |
| --- | --- | --- |
| 401. Do you engage in social activities     \| 1.Never \|  \| \| --- \| --- \| \| 2.Sometimes \|  \| \| 3. often \|  \| \| 4. very often \|  \| \| - 1. Not applicable \|  \| | 402. Do you participate in:   \|  \| 1. Yes \| 2. No \| \| --- \| --- \| --- \| \| 1. Religious functions \|  \|  \| \| 1. Elderly clubs \|  \|  \| \| 1. Local ceremonies (e.g. introduction ceremonies \|  \|  \| \| 1. Visiting friends recreational activities (e.g. ) \|  \|  \| \| 1. Charity walks \|  \|  \| \| 1. Social gatherings (e.g. drinking gatherings) \|  \|  \| \| 1. Attending community meetings \|  \|  \| \| 88. Others (specify) \|  \|  \| | 403. How often do you engage in this social activity   \| 1.Daily \|  \| \| --- \| --- \| \| 2. Weekly \|  \| \| 3. Monthly \|  \| \| 4. Quarterly \|  \| \| 5.Annually \|  \| \| 88. Others specify \|  \| |
| 404. During the last six months**,** how often did you go to a religious activity such as church, mosque or synagogue?   \| 1.Daily \|  \| \| --- \| --- \| \| 2. Weekly \|  \| \| 3. Monthly \|  \| \| 4. Quarterly \|  \| \| 88. Others specify \|  \| | 405. Did you attend these social functions in the last six months?   \|  \| 1. Yes \| 2. No \| \| --- \| --- \| --- \| \| 1. Wedding \|  \|  \| \| 2. Public meeting \|  \|  \| \| 3. burials/funerals \|  \|  \| \| 88. Other (specify) \|  \|  \| | 406. What hinders you from engageing in social activities?   \| 1. I am not a member \|  \| \| --- \| --- \| \| 1. Not invited \|  \| \| 1. Health problems \|  \| \| 1. I do not have to \|  \| \| 1. money \|  \| \| 88. Others(specify) \|  \| |
| 407. Do you feel you are discriminated against from social activities because of your health condition?   \| 1. Yes \|  \| \| --- \| --- \| \| 2. No \|  \| | 408. If there is any problem in the community, do people call you to participate in resolving it?   \| 1. Yes \|  \| \| --- \| --- \| \| 2. No \|  \| | 409. How satisfied are you with the way you spend your time?   \| 1. Dissatisfied \|  \| \| --- \| --- \| \| 1. Satisfied \|  \| \| 1. Very satisfied \|  \| |

**SECTION 500: PHYSICAL ACTIVITY**

**Now, I want to ask you about physical activities you do and how you participate in your community**

| **Question** | **Question** | **Question** |
| --- | --- | --- |
| 501. Do you engage in physical activities?   \| 1. A little \|  \| \| --- \| --- \| \| 1. Moderate \|  \| \| 1. Mostly \|  \| \| 1. completely \|  \| \| 1. Not applicable \|  \| | 502. What is your main activity   \| 1.Paid \|  \| 3. voluntary \|  \| \| --- \| --- \| --- \| --- \| \| 2. Unpaid \|  \| 88. Others (specify) \|  \| | 503. Are you satisfied with the main activity you do?   \| 1. Yes \|  \| \| --- \| --- \| \| 2. No \|  \| |
| 504. During the last seven days, have you done any of :   \|  \| 1. Yes \| 2. No \| \| --- \| --- \| --- \| \| 1. House work \|  \|  \| \| 1. Heavy work \|  \|  \| \| 1. Heavy gardening \|  \|  \| \| 1. Light gardening \|  \|  \| \| 1. Lift a heavy object \|  \|  \| \| 1. Stand for a long time \|  \|  \| | 505. Over the past 7 days, how often did you participate in sitting activities such as reading, or doing handcrafts? | 506. How satisfied are you with your capacity for work?   \| 1. A little \|  \| \| --- \| --- \| \| 1. Moderate \|  \| \| 1. Mostly \|  \| \| 1. completely \|  \| |
| 507. How would you rate your ability to work?   \| 1. Very poor \|  \| \| --- \| --- \| \| 1. Poor \|  \| \| 1. Good \|  \| \| 1. Very good \|  \| | 508. What prevents you from working? | 509. Do you participate in doing community activities?   \| 1. A little \|  \| \| --- \| --- \| \| 1. Moderate \|  \| \| 1. Mostly \|  \| \| 1. completely \|  \| \| 1. Not at all \|  \| |
| 510. What community activities do you engage in? | 511. Does your community recognize you?   \| 1. A little \|  \| \| --- \| --- \| \| 1. Moderate \|  \| \| 1. Mostly \|  \| \| 1. completely \|  \| \| 1. Not at all \|  \| | 512. In what ways are you recognized? |
| 513. If not why are you not recognized in your community? | 514. Overall, are you satisfied with the way you spend your time?   \| 1. A little \|  \| \| --- \| --- \| \| 1. Moderate \|  \| \| 1. Mostly \|  \| \| 1. completely \|  \| \| 1. Not at all \|  \| | 515 If no, why? |

**SECTION 600: PHYSICAL HEALTH**

Now, I am going to ask you questions on your mobility status and activities of daily living

| **Question** | **Question** | **Question** |
| --- | --- | --- |
| 601. I have difficulties in seeing   \| 1. Strongly agree \|  \| \| --- \| --- \| \| 1. Agree \|  \| \| 1. Neutral \|  \| \| 1. Disagree \|  \| \| 1. Strongly disagree \|  \|   Skip 602-603 if answer is 4 or 5 | 602. Does this difficult disturb you?   \| 1. Yes \|  \|  \| \| --- \| --- \| --- \| \| 2. No \|  \|  \| | 603. For how long have you been with the problem?  Answer in years |
| 604. I have difficulties in hearing   \| 1. Strongly agree \|  \| \| --- \| --- \| \| 1. Agree \|  \| \| 1. Neutral \|  \| \| 1. Disagree \|  \| \| 1. Strongly disagree \|  \|   Skip 605-606 if answer is 4 or 5 | 605. Does this difficult disturb you?   \| 1. Yes \|  \|  \| \| --- \| --- \| --- \| \| 2. No \|  \|  \| | 606. For how long have you been with the problem?  Answer in years |
| 607. I have difficulties in remembering or concentrating   \| 1. Strongly agree \|  \| \| --- \| --- \| \| 1. Agree \|  \| \| 1. Neutral \|  \| \| 1. Disagree \|  \| \| 1. Strongly disagree \|  \|   Skip to 608-609 if answer is 4 or 5 | 608. Does this difficult disturb you?   \| 1. Yes \|  \|  \| \| --- \| --- \| --- \| \| 2. No \|  \|  \| | 609. For how long have you been with the problem?  Answer in years |
| 610. I have difficulties in moving on my own.   \| 1. Strongly agree \|  \| \| --- \| --- \| \| 1. Agree \|  \| \| 1. Neutral \|  \| \| 1. Disagree \|  \| \| 1. Strongly disagree \|  \| | 611. What causes this difficulty?   \| 1. Pain in joints \|  \| \| --- \| --- \| \| 2. Body weakness \|  \| \| 3. Disability \|  \| \| 88. Others (specify) \|  \| | 612. Does this difficulty bother you?   \| 1. Yes \|  \|  \| \| --- \| --- \| --- \| \| 2. No \|  \|  \| |
| 613. For how long have you had this problem?  Years   \|  \| \| --- \| | 614. How often do you take a walk outside your home for fun, excise etc. in a week?   \| 1. Once \|  \| \| --- \| --- \| \| 2. More than twice \|  \| \| 3. I did not walk at all \|  \| | 615. What is the longest distance you have walked in the past month (Km)? |
| 616. How well are you able to get around?   \| 1. Poor \|  \| \| --- \| --- \| \| 2. Good \|  \| | 617. To what extent do any difficulties in movement affect your way of life? | 618. How satisfied are you with your ability to move around? |
| 619. How important to you is it to be able to move around? | 620. What type of transport means do you use?   \| 1. Public \|  \| \| --- \| --- \| \| 2. Private \|  \| \| 3. others (specify) \|  \| | 621. If you use private transport, which type?   \| 1. Car hire \|  \| \| --- \| --- \| \| 2. My car \|  \| \| 3. Boda Boda \|  \| \| 3 Bicycle \|  \| \| 88. others (specify) \|  \| |
| 622. I always have difficulties with feeding myself   \| 1. Strongly agree \|  \| \| --- \| --- \| \| 1. Agree \|  \| \| 1. Neutral \|  \| \| 1. Disagree \|  \| \| 1. Strongly disagree \|  \|   Skip 623 if answer is 4 or 5 | 623. If yes how do you solve it? | 624. I always have difficulties while taking a bath or shower.   \| 1. Strongly agree \|  \| \| --- \| --- \| \| 1. Agree \|  \| \| 1. Neutral \|  \| \| 1. Disagree \|  \| \| 1. Strongly disagree \|  \|   Skip 625 if answer is 4 or 5 |
| 625. If yes how do you solve it? | 626. I always have difficulties while dressing   \| 1. Strongly agree \|  \| \| --- \| --- \| \| 1. Agree \|  \| \| 1. Neutral \|  \| \| 1. Disagree \|  \| \| 1. Strongly disagree \|  \|   Skip 607 if answer is 4 or 5 | 627. 622. If yes how do you solve it? |
| 628. Are you able to move from your bed to a chair?   \| 1. Yes \|  \|  \| \| --- \| --- \| --- \| \| 2. No \|  \| **Skip to Q. 701** \| | 629. 622. If yes how do you solve this challenge? |  |

**SECTION 700: BODY PAIN, SLEEP AND PERCEIVED QUALITY OF LIFE**

Now, I am going to ask you questions on your health and how you perceive your quality of life.

| **Question** | **Question** | **Question** |
| --- | --- | --- |
| 701. I have been ill in the past one month.   \| 1. strongly agree \|  \| \| --- \| --- \| \| 2. Agree \|  \| \| 3. Neutral \|  \| \| 4. Disagree \|  \| \| 5. Strongly disagree \|  \| | 702. Are you currently suffering from any of the following diseases that result into body pain?   \| 1. Diabetes \|  \| \| --- \| --- \| \| 2. High blood pressure \|  \| \| 3. Heart diseases \|  \| \| 4. Asthma \|  \| \| 5.Cancer \|  \| \| 6.Bronchitis \|  \| \| 7.HIV/AIDS \|  \| \| 8.Eating disorders \|  \| \| 88.Others (specify \|  \| | 703. Is it a professional medical worker like a doctor who told you that you have any of the diseases in question 702?   \| 1. Yes \|  \| \| --- \| --- \| \| 2. No \|  \| |
| 704. What other health conditions cause pain in your body? | 705. During the past three months, have you experienced pain around the joints (like arms, hands, legs or feet because of any illness or injury?   \| 1. Very severe \|  \| \| --- \| --- \| \| 2. Severe \|  \| \| 3. Mild no pain at all \|  \| \| 4.Not at all \|  \| | 706 How much joint pain have you had during the last three months?   \| 1. Very severe \|  \| \| --- \| --- \| \| 2. Severe \|  \| \| 3. Mild no pain at all \|  \| \| 4.Others (specify) \|  \| |
| 707. Are you currently taking pain medication?   \| 1. Yes \|  \|  \| \| --- \| --- \| --- \| \| 2. No \|  \| **Skip to Q 710** \| | 708. How often do you take pain medication?   \| 1. Daily \|  \| \| --- \| --- \| \| 2. Weekly \|  \| \| 3. Monthly \|  \| \| 88.Others (specify) \|  \| | 709. Is your pain reduced by the medication you take?   \| 1. Yes \|  \| \| --- \| --- \| \| 2. No \|  \| |
| 710. Do you use other measures to control your pain?   \| 1. Yes \|  \|  \| \| --- \| --- \| --- \| \| 2. No \|  \| **Skip to Q 722** \| | 711. What other measures do you use to control pain? | 712. Is your pain controlled now   \| 1. Yes \|  \| \| --- \| --- \| \| 2. No \|  \| |
| 713. Given the degree to which your pain is reduced, do you think something more should be done to help control your pain?   \| 1. Yes \|  \| \| --- \| --- \| \| 2. No \|  \| | 714. Do you worry about your pain or discomfort?   \| 1. Yes \|  \| \| --- \| --- \| \| 2. No \|  \| | 715. Is it difficult for you to handle any pain?   \| 1. Yes \|  \| \| --- \| --- \| \| 2. No \|  \| |
| 716. Does you physical pain prevent you from doing what you need to do?   \| 1. Yes \|  \| \| --- \| --- \| \| 2. No \|  \| | 717. Do you worry about possible future (physical) pain?   \| 1. Yes \|  \| \| --- \| --- \| \| 2. No \|  \| | 718. Below are activities that you may have participated in recently. Please indicate YES or NO to show whether you have done the activity in the last one month   \| 1. Gone for shopping \|  \| \| --- \| --- \| \| 2. Prepared a meal \|  \| \| 3. Done the laundry \|  \| \| 4.Rode a bicycle \|  \| \| 5.Exercised in health club/outside \|  \| |
| 719. In the **past four weeks**, I have had difficulties with brushing my teeth.   \| 1. Strongly agree \|  \| \| --- \| --- \| \| 1. Agree \|  \| \| 1. Neutral \|  \| \| 1. Disagree \|  \| \| 1. Strongly disagree \|  \| | 720. I easily get tired even when I have not done any work.   \| 1. Strongly agree \|  \| \| --- \| --- \| \| 1. Agree \|  \| \| 1. Neutral \|  \| \| 1. Disagree \|  \| \| 1. Strongly disagree \|  \| | 721. How much are you bothered by fatigue?   \| 1. Not at all \|  \| \| --- \| --- \| \| 2. A little \|  \| \| 3. A moderate amount \|  \| \| 4. Very much \|  \| \| 5. An extreme amount \|  \| |
| 722. I have enough energy for everyday life.   \| 1. Strongly agree \|  \| \| --- \| --- \| \| 1. Agree \|  \| \| 1. Neutral \|  \| \| 1. Disagree \|  \| \| 1. Strongly disagree \|  \| | 723. How satisfied are you with the strength that you have?   \| 1. Not at all \|  \| \| --- \| --- \| \| 2. A little \|  \| \| 3. Moderate \|  \| \| 4. Very much \|  \| | 724. I have difficulties with sleeping   \| 1.Strongly agree \|  \| \| --- \| --- \| \| 2. Agree \|  \| \| 3. Neutral \|  \| \| 4. Disagree \|  \| \| 5. Strongly disagree \|  \| |
| 725. To what extent does any sleep problems worry you?   \| 1. A little \|  \| \| --- \| --- \| \| 1. Moderate \|  \| \| 1. Mostly \|  \| \| 1. completely \|  \| \| 1. Not at all \|  \| | 726. on average, how long do you sleep per night?   \|  \| \| --- \| | 727. In general, how would you rate the quality of your life as a whole?   \| 1. Good \|  \|  \| \| --- \| --- \| --- \| \| 2. Poor \|  \| **Skip to Q 728** \| |
| 728. If poor, why? |  |  |

**SECTION 800: INTIMACY**

**Now, I want to ask you issues about companionship, love and sexuality**

| **Question** | **Question** | **Question** |
| --- | --- | --- |
| 801. I have opportunities to love other people.   \| 1. Strongly agree \|  \| \| --- \| --- \| \| 1. Agree \|  \| \| 1. Neutral \|  \| \| 1. Disagree \|  \| \| 1. Strongly disagree \|  \| | 802. If not why? | 803. I am satisfied with my companionship in life.   \| 1. Strongly agree \|  \| \| --- \| --- \| \| 1. Agree \|  \| \| 1. Neutral \|  \| \| 1. Disagree \|  \| \| 1. Strongly disagree \|  \| |
| 804. If not, why? | 805. I sometimes engage in sex.   \| 1. Strongly agree \|  \| \| --- \| --- \| \| 1. Agree \|  \| \| 1. Neutral \|  \| \| 1. Disagree \|  \| \| 1. Strongly disagree \|  \| | 806. If not married, do you use protection? |
| 807. I nolonger engage in sexual relations.   \| 1. Strongly agree \|  \| \| --- \| --- \| \| 1. Agree \|  \| \| 1. Neutral \|  \| \| 1. Disagree \|  \| \| 1. Strongly disagree \|  \| | 808. If 1 or 2, why? |  |

**THANK YOU VERY MUCH FOR YOUR TIME**
